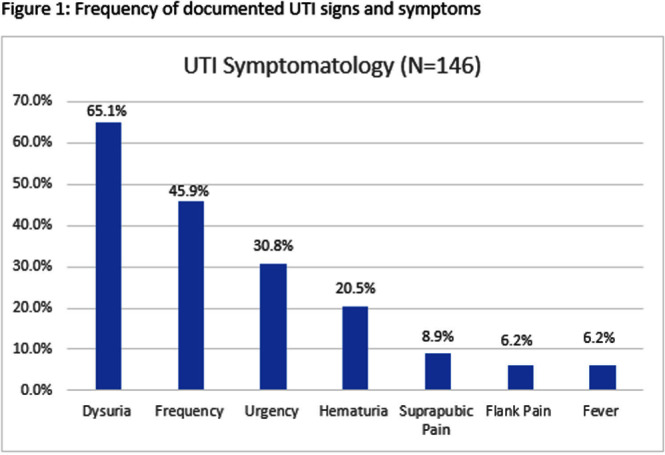# UTI Symptomatology and Antibiotic Prescribing among US Veterans Seen in Outpatient Clinics

**DOI:** 10.1017/ash.2024.214

**Published:** 2024-09-16

**Authors:** Geneva Wilson, Ravyn Jackson, Sara Abdelrahim, Taissa Bej, Robin Jump, Charlesnika Evans

**Affiliations:** Edward Hines Jr. VA Hospital; Department of Veterans Affairs; VA Cleveland Healthcare System; VA Northeast Ohio Healthcare System; VA Pittsburgh Healthcare System; Northwestern University, Feinberg School of Medicine

## Abstract

**Background:** Infectious Diseases Society of America guidelines recommend antibiotic prescribing for urinary tract infections (UTIs) when there is a positive culture and signs and symptoms of infection. Despite these guidelines, prescribing for asymptomatic bacteriuria remains prevalent. We conducted a chart review of UTI outpatient encounters to determine the prevalence of antibiotic prescribing as well as patient and provider factors associated with inappropriate prescribing for UTIs. **Methods:** Patients who were seen at any Department of Veterans Affairs (VA) outpatient clinic with a positive urine culture from 1/1/2019-12/31/2022 were evaluated for inclusion. Exclusion criteria were pregnancy, neutropenia, neurogenic bladder, spinal cord injury/disorder, chronic kidney disease stage III and above, and those undergoing urologic surgical procedures within 7 days. Inappropriate prescribing was defined as an antibiotic prescription given for UTI treatment when no signs or symptoms of infection were recorded during the patient encounter. Chi-square, Fisher’s exact and t-tests were used to evaluate the association between patient and provider characteristics and antibiotic prescribing. **Results:** Among 341 visits, most patients were male (70%), White (40%), older (mean age of 65.8 ± 15.9 years) and treated at an urban facility (57%). Antibiotics were prescribed for 67% (229/341) of visits. Of the 229 antibiotic courses prescribed, 119 (52%) were appropriate; issued to patients with > = 1 sign or symptom consistent with a urinary tract infection. The most common symptom recorded was dysuria, followed by frequency, urgency, and hematuria (Figure 1). The remaining 110 (48%) antibiotic prescriptions were inappropriate; given to patients without documented UTI-related signs or symptoms. The proportion of inappropriate prescribing was higher among advanced practice practitioners (39/56; 69%) compared to physicians (68/113; 60%; P < 0 .0001). Prescribing of an antibiotic did not differ by gender (p-value=0.3779), race (p-value=0.3972), age (p-value=0.7461) or urban versus rural geography (p-value=0.3647). Discussion: In outpatient clinics, nearly half of antibiotics prescribed to patients with a positive urine culture occurred in the absence of documented of signs or symptoms of a UTI. These results suggest that interventions to improve antibiotic use for UTI-related concerns in the outpatient setting should address UTI-related signs and symptoms as well as asymptomatic bacteriuria. Advanced practice practitioners were more likely to prescribe without documentation of relevant signs or symptoms than physicians. Improving meaningful documentation about the presence or absence of signs and symptoms of a UTI may help reduce inappropriate antibiotic prescriptions in the outpatient setting.

**Disclosure:** Robin Jump: Research support to my institution from Merck and Pfizer; Advisory boards for Pfizer

**Figure:**